# Supplementary figures and images for: A comparison of health care worker-collected foam and polyester nasal swabs in convalescent COVID-19 patients
Source: PLoS One. 2020 Oct 27;15(10):e0241100. doi: 10.1371/journal.pone.0241100 (PMC7591034; doi:10.1371/journal.pone.0241100)

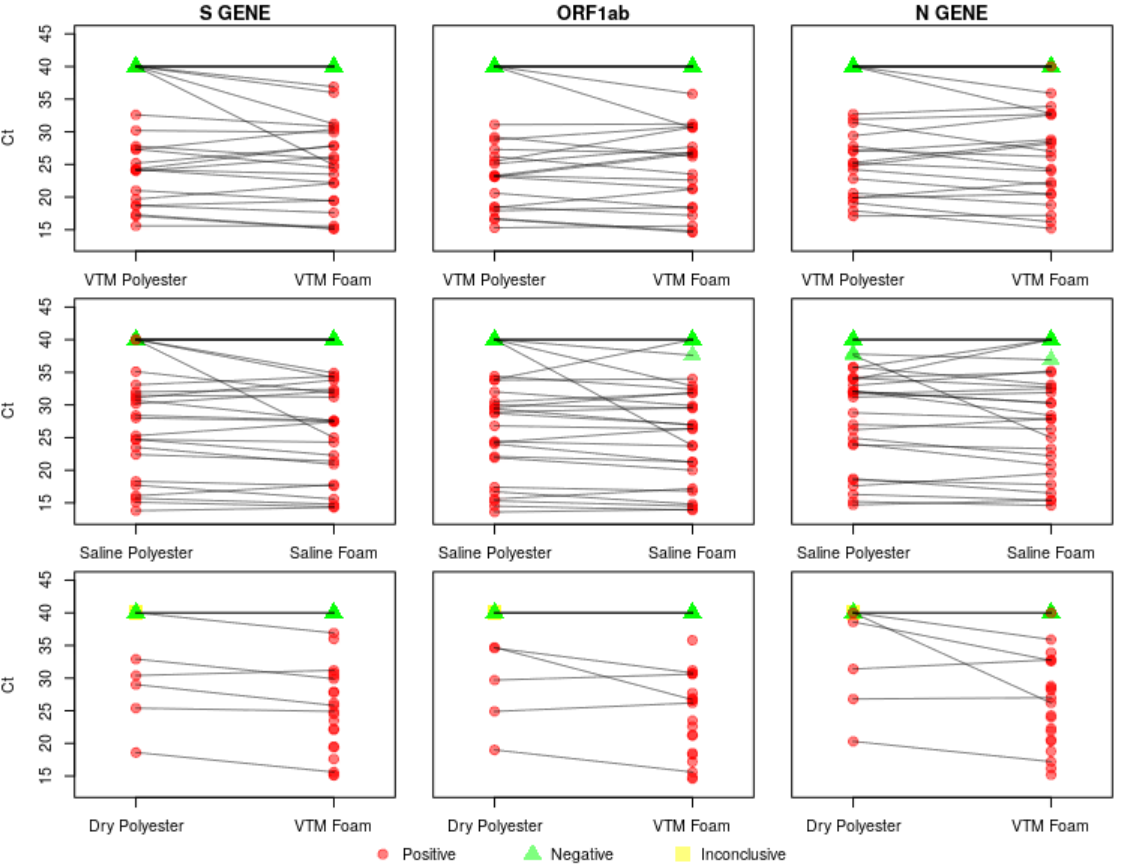

Supplement: S1 Fig — Paired Ct plots showing the polyester and foam Ct values for each transport media and target gene combination considered for swabs from patients less than 10 days from symptom onset at the time of sample collection. Swabs collected at the same visit are connected by a black line. (TIF) [file pone.0241100.s001.tif]

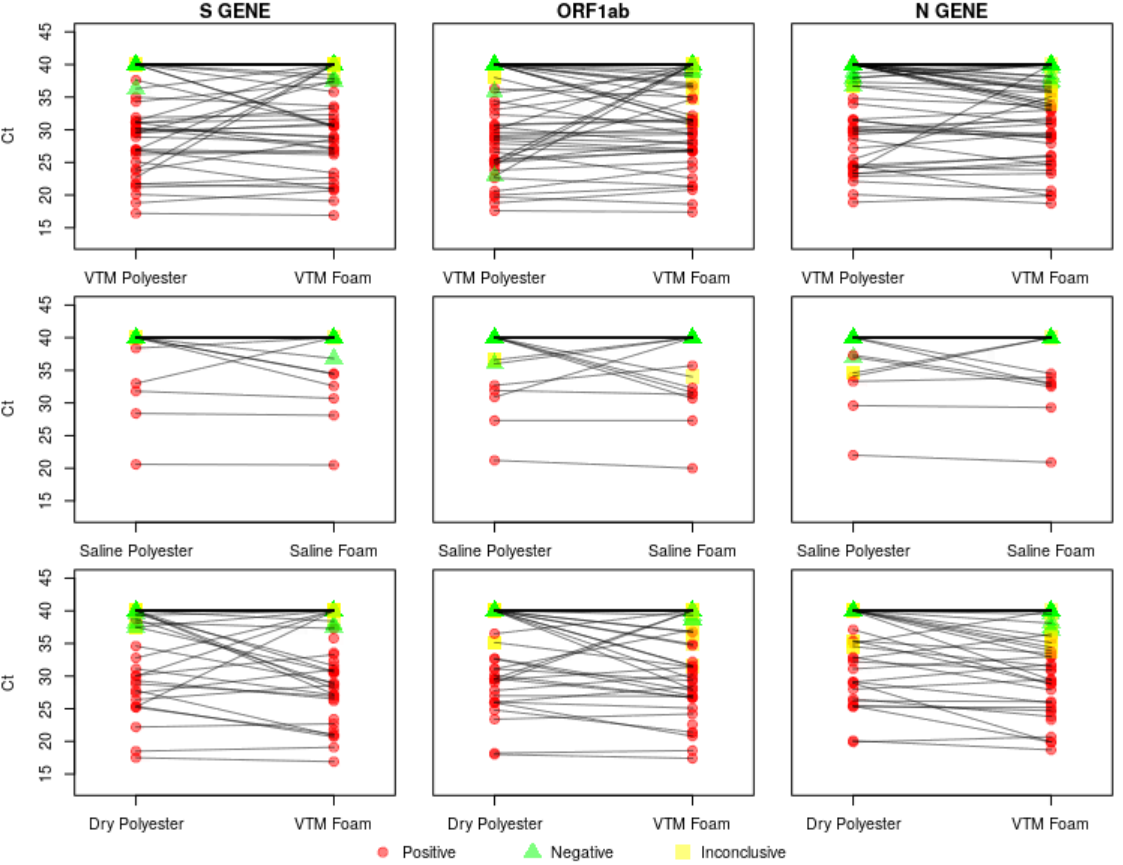

Supplement: S2 Fig — Paired Ct plots showing the polyester and foam Ct values for each transport media and target gene combination considered for swabs from patients at least 10 days from symptom onset at the time of sample collection. Swabs collected at the same visit are connected by a black line. (TIF) [file pone.0241100.s002.tif]
