# Supplementary material for: A comparison of health care worker-collected foam and polyester nasal swabs in convalescent COVID-19 patients
Source: PLoS One. 2020 Oct 27;15(10):e0241100. doi: 10.1371/journal.pone.0241100 (PMC7591034; doi:10.1371/journal.pone.0241100)
Supplement: Attachment — Submitted filename: Response to Reviewers_9_15.docx [file pone.0241100.s003.docx]

The authors would like to thank the reviewers and editor for their careful review and consideration of our manuscript titled “A comparison of health care worker-collected foam and polyester nasal swabs in convalescent COVID-19 patients”. Below we provide a point by point response to the reviewer comments below:

- Please discuss if different volume of VTM or saline used, or normalization of Ct values would affect the results (Reviewer #1).

Yes, using different sample volumes and applying mathematical corrections is not ideal and could affect the results. However, considering the urgent need for these studies, the standard 1-3 mL volumes used for clinical sample collection, and the valid mathematical approach for performing fold change calculations on qPCR, we felt normalization of the data was the best approach. For clarification, we have added text to the methods section and to the discussion.

Methods:

“Because of the volumetric differences among the three types of collection and/or elution media, mathematical adjustments to some Ct values were made in order to account for the differences in dilution of the various swab samples. By scaling (i.e., normalizing) Cts for both the 2 mL saline samples and the 3 mL VTM samples to match the 1 mL sample volume (VTM or 1 mL PBS dry swab elution), the sensitivity comparisons among the three sample collection volumes are more legitimate than using unnormalized data. Values were normalized as follows: to account for the 2-fold dilution difference between saline (2 mL) and dry polyester (1 mL PBS) or foam (1 mL VTM), 1 Ct was subtracted from all qPCR results for the former; similarly, to account for the 3-fold sample volume difference between the 3 mL VTM polyester swab samples and dry polyester (1 mL PBS) or foam (1 mL VTM), 1.585 Ct was subtracted from the former. Note that the specific Ct adjustments follow the formula 2-dCt=F, where dCt is the necessary Ct adjustment and F is the fold increase in volume. This formula assumes the assays perform at approximately 100% PCR efficiency during the geometric phase of amplification.”

Discussion:

“Although final Ct values were adjusted for varying amounts of transport media, imprecision in these adjustments could confound data interpretation; specifically, comparisons between the mathematically corrected samples (2 mL and 3 mL) and the non-corrected samples (1 mL) present the greatest risk for error.”

- Provide basic clinical information of the two cohorts (Reviewer #2).

Thank you for the suggestion. We have added in a new table one with basic clinical/demographic information available on the two cohorts.

- Please present the number of samples collected by day of visit group

These values can now be found in the expanded version of Tables 2 (previously Table 1).

- Tables 1-3 have pooled samples collected at different days after diagnosis. However, viral shedding decreased over time and hence test sensitivity is unlikely constant over sampling dates. Please further assess the sensitivity by the time of sampling.

Table 2 (previously called Table 1) has been expanded to breakout the results by the three different visits. Note that Tables 3 and 4 contain results which were collected at a single visit, so no similar table breakout was necessary/possible.

- The authors have assessed sensitivity of different sampling methods. Could the authors explain why specificity was not assessed?

When estimating sensitivity we assume that all positives were true positives. The primary advantage of this approach is that it allows for the estimation of sensitivity without assuming a single method is a gold standard “truth”. The disadvantage of our approach is that assuming all positives are true positives implies that all methods have a specificity of 100%. One could alternatively treat a single method (e.g. foam VTM) as the truth and compare the other methods when estimating sensitivity. This approach also involves strong assumptions and, while it allows for the estimation of both sensitivity and specificity of the non-reference methods, it implies 100% sensitivity and specificity of the reference method. Based on these tradeoffs, we chose to treat any positive as a true positive even though it precludes accurate specificity estimation.

In addition to the edits made in response to reviewer comments we have made some additional changes to the description of the procedures. These changes were made so that the manuscript accurately describes how the study was carried out in practice. The sample collection procedures are now more accurately and clearly described in the manuscript. We have also changed the method used to calculate the 95% confidence intervals from the Normal approximation to the Wilson Score method, which has been shown to have better coverage. This change was motivated by the need for more stable confidence interval estimates given the low sample sizes, especially when broken out by visit.
