# Supplementary material for: A comparison of health care worker-collected foam and polyester nasal swabs in convalescent COVID-19 patients
Source: PLoS One. 2020 Oct 27;15(10):e0241100. doi: 10.1371/journal.pone.0241100 (PMC7591034; doi:10.1371/journal.pone.0241100)
Supplement: Attachment — Submitted filename: Response to Reviewers Round 2.docx [file pone.0241100.s004.docx]

The authors would like to thank the reviewers and editor for their careful review and consideration of our manuscript titled “A comparison of health care worker-collected foam and polyester nasal swabs in convalescent COVID-19 patients”. Below we provide a point by point response to the reviewer comments below:

- line 249-250 in the discussion: there is a sentence fragment that needs to be removed or completed.

Thank you for catching the error. The sentence fragment has been removed in the resubmitted manuscript.

- Although I will not "require" it, it would be helpful to provide p-values for comparisons across swab types and/or storage.

We have now provided p-values from a Fisher’s Exact test for the three polyester versus foam sensitivity comparisons. The p-values are included in the main text and in Tables 2-4. In Table 2, p-values were not included for the visit specific sensitivity comparisons due to small sample sizes. Two sentences have been added to the Statistical analysis section to explain methods used for p-value calculations.

No additional changes were made to the manuscript. We again thank the reviewers for their constructive feedback which has led to a much improved manuscript.
